# A natural and readily available crowding agent: NMR studies of proteins in hen egg white

**DOI:** 10.1002/prot.22967

**Published:** 2010-12-13

**Authors:** Gabriel Martorell, Miquel Adrover, Geoff Kelly, Piero Andrea Temussi, Annalisa Pastore

**Affiliations:** 1 Serveis Científico-Tècnics, Universitat de les Illes BalearsPalma de Mallorca, Spain; 2 MRC National Institute for Medical ResearchThe Ridgeway, London NW7 1AA, United Kingdom; 3 Departament de Química, Universitat de les Illes BalearsPalma de Mallorca, Spain; 4 Department of Chemistry, Università di Napoli Federico IIvia Cinthia, Napoli 80126, Italy

**Keywords:** confinement, stability, folding, aggregation, cytoplasm

## Abstract

*In vitro* studies of biological macromolecules are usually performed in dilute, buffered solutions containing one or just a few different biological macromolecules. Under these conditions, the interactions among molecules are diffusion limited. On the contrary, in living systems, macromolecules of a given type are surrounded by many others, at very high total concentrations. In the last few years, there has been an increasing effort to study biological macromolecules directly in natural crowded environments, as in intact bacterial cells or by mimicking natural crowding by adding proteins, polysaccharides, or even synthetic polymers. Here, we propose the use of hen egg white (HEW) as a simple natural medium, with all features of the media of crowded cells, that could be used by any researcher without difficulty and inexpensively. We present a study of the stability and dynamics behavior of model proteins in HEW, chosen as a prototypical, readily accessible natural medium that can mimic cytosol. We show that two typical globular proteins, dissolved in HEW, give NMR spectra very similar to those obtained in dilute buffers, although dynamic parameters are clearly affected by the crowded medium. The thermal stability of one of these proteins, measured in a range comprising both heat and cold denaturation, is also similar to that in buffer. Our data open new possibilities to the study of proteins in natural crowded media. Proteins 2011. © 2010 Wiley-Liss, Inc.

## INTRODUCTION

Most *in vitro* studies of biological macromolecules are performed in dilute solutions where the interactions among a few different types of molecules are diffusion limited. However, in living systems, several macromolecules, different from those we are interested in, are present as soluble species and structural arrays at very high-total concentrations.^1^ Natural media in living cells are, thus, often referred to as “crowded”^2^ or “confining”^3^ rather than “concentrated,” referring to the heterogeneity of proteins present. Numerous measurements inside cells confirm that their interior is not well described by the dilute solution paradigm.^4^ Crowding and confinement can for instance influence protein aggregation, presumably by altering the kinetics and the equilibrium parameters of aggregation equilibria or even by affecting protein stability.^5^,^6^

Several attempts have been made over the last few years to study biological macromolecules directly in natural crowded environments. The effect of crowded environments has been widely studied using several potential crowders: proteins, polysaccharides, and synthetic polymers.^7^ These studies have been very helpful in clarifying the limits of the influence of crowded environments from a physicochemical point of view. However, it is always difficult to extrapolate physicochemical studies to actual behavior *in vivo* if the model system is rather far frombiology.

A way to study crowding closer to natural environments is the possibility of measuring nuclear magnetic resonance (NMR) spectra of proteins directly overexpressed in intact bacterial cells.^8^ Some of these studies are really spectacular but, alas, the approach does not seem to be generally applicable to the system of choice. A completely different approach was proposed by Selenko and Wagner.^9^ They used intact oocytes from *Xenopous levis* as a confined, crowded, natural medium, and recorded NMR spectra of isotopically labeled proteins injected into cells. Brilliant as it is, this approach may be limited by its intrinsic technical difficulties.

We have looked for a simple natural medium, with all the features of in-cell crowding that could be used by any researcher without difficulty and inexpensively. We converged to the albumen of avian eggs, for example, hen egg white (HEW). This protective medium is composed mainly of about 40 soluble proteins (http://en.wikipedia.org/wiki/Egg_white). The most abundant proteins are ovalbumin, ovotransferrin, ovomucoid, ovoglobulin, ovomucin, and lysozyme. The high percentage of these macromolecules (more than 12% by weight) confers to HEW all the characteristics of a crowded environment. Recent findings on the peculiarity of protein crowders with respect to synthetic polymers^7^,^10^ emphasize the role of proteins, with respect to other macromolecules, in natural media.

Before using HEW as a medium for the study of diffusion-limited protein binding and self-aggregation, we need to characterize the behavior of proteins in this medium and study whether it interferes with protein structure, protein stability and/or NMR spectral features. Here, we present a study of the properties of two model proteins in ovo, in terms of structural integrity, dynamics and thermal stability. To study the feasibility of HEW as a medium suitable for NMR studies, we used members of the frataxin family, which comprises small proteins (11–13 kDa) related to the neurodegenerative disease Friedreich ataxia. This family has been thoroughly characterized in our laboratory.^11^–^13^ In addition to their medical relevance, frataxins are interesting from the point of view of thermodynamic stability, which greatly varies according to the ortholog. The *E. coli* and *H. sapiens* orthologs unfold at temperatures of about 55–65°C, whereas the *S. cerevisiae* ortholog (Yfh1) has two melting points at about 5°C and around 30°C.^14^ This latter circumstance makes this protein a prototypical example of a natural protein that undergoes cold denaturation at temperatures around the water freezing point and offers the unique possibility to measure the whole stability curve of a protein in HEW: although undiluted HEW coagulates at about 60°C, this temperature is well above the heat denaturation temperature of Yfh1.

Our studies provide strong evidence that HEW is a suitable medium for crowding studies, which can uniquely exploit NMR techniques for the study of proteins in natural environments.

## MATERIALS AND METHODS

### Protein production

Pure ovalbumin was purchased from Sigma Aldrich (UK). Recombinant *E. coli* CyaY and *S. cerevisiae* Yfh1 were produced as previously described in detail.^11^,^15^ In short, CyaY (Swiss-Prot:P27838) was cloned into a pET-derived plasmid vector as a His-tagged glutathione-S-transferase (GST) fusion protein comprising a tobacco etch virus protease cleavage site, which leaves a short N-terminal GlyAla tag. For Yfh1, we used a pET11A expression vector in which residues 52–174, which correspond to the mitochondrial mature form of the protein, had been subcloned. This vector was a generous gift of Stemmler (Wayne State University). ^15^N labeled and ^15^N labeled samples were produced by growing the bacteria in minimal medium using ammonium sulfate as the sole source of nitrogen. The proteins were expressed in *Escherichia coli* BL21-(DE3) cells grown at 37°C, induced with in 1 m*M* IPTG for 6 h, lysed with a French press and sonicated. CyaY was first subjected to affinity chromatography (glutathione-*S*-Sepharose) and then further purified by gel filtration chromatography on a Superdex G75 16/60 column (Pharmacia). Yfh1 was purified by two ammonium sulfate precipitation steps with a 40% cut to precipitate contaminating proteins and a 65% cut to precipitate Yfh1. After dialysis, the protein was subjected to anion exchange chromatography using a Pharmacia Q-Sepharose column with a gradient to 1*M* NaCl, followed by a Pharmacia phenyl-Sepharose column with a decreasing 1*M* ammonium sulfate gradient. The EDTA containing protease inhibitor cocktail (Roche) was added before the cells were lysed and at each purification step. EDTA and salts were removed by dialysis before concentration of the protein. Protein purity and identity were checked using SDS-PAGE and mass spectrometry.

### Sample preparation in egg

Several types of chicken egg whites and one from quail egg were compared and evaluated in relationship to their features, notably pH, in relation to ageing. Samples of egg white, to be used as medium in NMR experiments, were taken either from commercial chicken eggs or from free-range chicken eggs collected in two villages of Mallorca (Son Sardina and Calonge). Mechanical separation from yolk and subsequent filtering with cheesecloth allowed elimination of chalazae and membranes, to retain only the thin portion of HEW. Samples of HEW were prepared and used immediately before NMR measurements. Lyophilized proteins were dissolved in about 0.5 mL of neat HEW together with 40 μL of D_2_O.

### NMR spectroscopy

NMR spectra were recorded on a Varian INOVA spectrometer and on a Bruker AVANCE, both operating at 600 MHz ^1^H frequency. Typically, measurements were carried out using protein (unlabeled or ^15^N uniformly labeled) concentrations of 0.3–0.5 m*M*. Water suppression was achieved by the WATERGATE pulse-sequence,^16^ HSQC experiments were used as described by Bax *et al*.^17^ The spectra were processed, zero-filled to the next power of two, or just analyzed using the NMRPipe program^18^ or SPARKY^19^ software. Baseline correction was applied when necessary.

^15^N *T*_1_, *T*_2_, and NOE NMR relaxation measurements were performed at 600 MHz and 25°C on approximately 0.25 m*M* samples. Both *T*_1_ data and *T*_2_ data were acquired with eight relaxation delays (10, 100, 200, 400, 700, 1000, 1500, and 2000 ms and 8.7, 17.4, 26.1, 34.8, 52.2, 69.6, 87.0, and 104.4 ms, respectively). Experimental steady-state NOE values were determined from the peak intensity ratios of amide signals obtained by recording interleaved 2D Watergate ^1^H-^15^N HSQC spectra with and without a proton saturation delay of 4 s and a repetition delay of 4.2 s. *T*_1_ and *T*_2_ relaxation times were obtained by fitting the data with a two-parameter single exponential decay function. The *T*_1_ and *T*_2_ values of residues that differ by more than two standard deviations from the mean value were not considered in the correlation time calculations.

*T*_1_ and *T*_2_ values for each residue were used to determine *R*_1_, *R*_2_ that in turn were fed to the r2r1_tm program (Palmer's group, http://www.hhmi.umbc.edu/toolkit/analysis/palmer/r2r1_tm.html) to determine the local correlation time for each residue. Averaging the obtained values, it was possible to determine τ_c_ values.

## RESULTS

### The spectral features of HEW are highly reproducible

We used both commercial and freshly laid eggs. When used within a few hours from deposition, eggs had a pH between 8.2 and 8.3. One week later, the pH reached values between 8.5 and 9.3, typically close to values of commercial eggs. A widely accepted explanation for this observation is that intact, freshly laid eggs contain a high concentration of CO_2_, which is then progressively lost during time (http://newton.ex.ac.uk/teaching/CDHW/egg/). After this initial variation which can be up to 1 pH unit, the pH drift is minimal.

**Figure 1:**
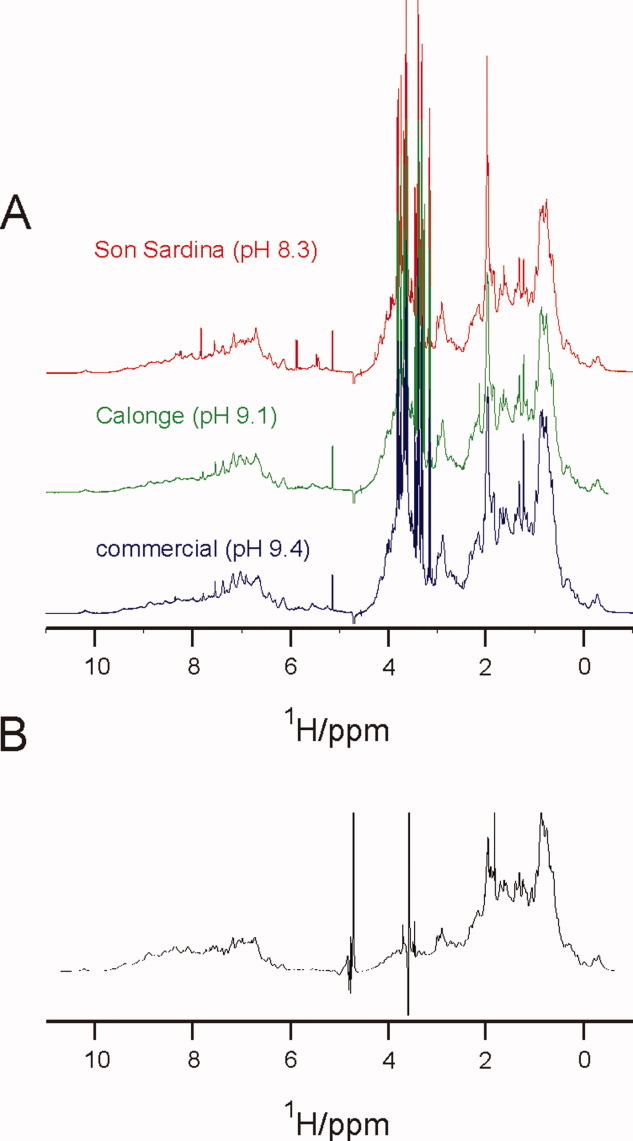
Comparison of 1D spectra of HEW and that of pure ovalbumin. **A**: Typical 1D ^1^H spectra of HEW of two free-range eggs and a commercial one. The sharp peaks between 3 and 4 ppm correspond to a complex mixture of carbohydrates. **B**: 1D ^1^H spectrum of pure ovabumin (1.33 m*M*) in 50 m*M* Tris-HCl buffer at pH 8.0.

Beyond this, the 1D proton spectra of eggs from different sources are well reproducible with overall very common features [Fig. 1(A)]. The spectra are dominated by the resonances of a single protein, which by comparison with the known HEW composition and literature spectra can be easily identified as ovalbumin, as its concentration in egg white is estimated approximately to 1.5 m*M* [Fig. 1(B)]. Additionally, in all spectra there are some sharp peaks centered around 3.8 ppm, which have a pattern typical of carbohydrate spectra.^20^ A few other sharp but less intense peaks in the aromatic region seem to be specific only for free-range eggs of a given source. Although the intensities and/or the chemical shifts of some of the small molecular weight peaks changed on aging, the envelops originating from HEW proteins did not. This finding indicates that there is no appreciable protein degradation or spectral alternations as necessary in view of the possible use of HEW as a medium for long NMR experiments.

### HEW does not affect the structural integrity of proteins

**Figure 2:**
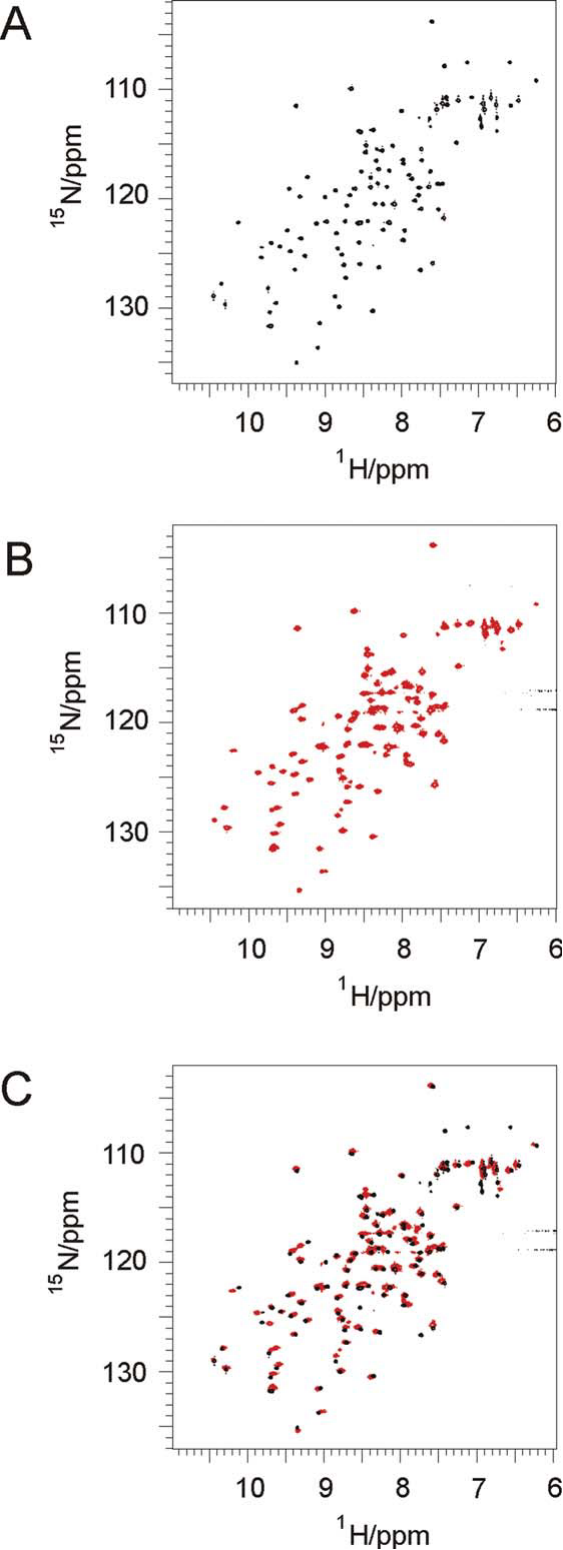
Comparison of the NMR spectra of Cyay in dilute-buffered solution and in HEW. **A**: ^1^H^15^N HSQC of CyaY in 25 m*M* Tris-HCl buffer at pH 8.3. **B**: ^1^H^15^N HSQC of CyaY in HEW. **C**: Superposition of the spectra of panels A and B.

We then tested whether CyaY, a small globular protein of 106 amino acids with an α/β-fold and metal binding properties, yields acceptable NMR spectra in HEW. Comparison of the HSQC spectra of CyaY in buffer and in HEW shows that all peaks indicative of properly folded proteins are retained in HEW, albeit significantly broadened [Figs. 2(A,B)]. The superposition of the two spectra confirms that there is no significant chemical shift perturbation in going from the dilute solution to the crowded environment [Fig. 2(C)].

The absence of significant chemical shift changes indicates that our natural environment did not produce any chemical modification, for example, phosphorylation, of the side chains of the probe protein. Interestingly, the only significant differences between the two spectra (inany case ≤0.1 ppm and 1 ppm in the proton and nitrogen dimensions, respectively) map to residues, which are known to be highly susceptible to the divalent cation content,^21^ thus, suggesting that HEW is anyway a more suitable model system of the in cell situation than any nonspecialized buffer.

### Protein flexibility in HEW

To better characterize the properties of CyaY in HEW we measured typical dynamical parameters by NMR ^15^N relaxation experiments. This technique has proven to be very successful in providing information about molecular internal motions.

**Figure 3:**
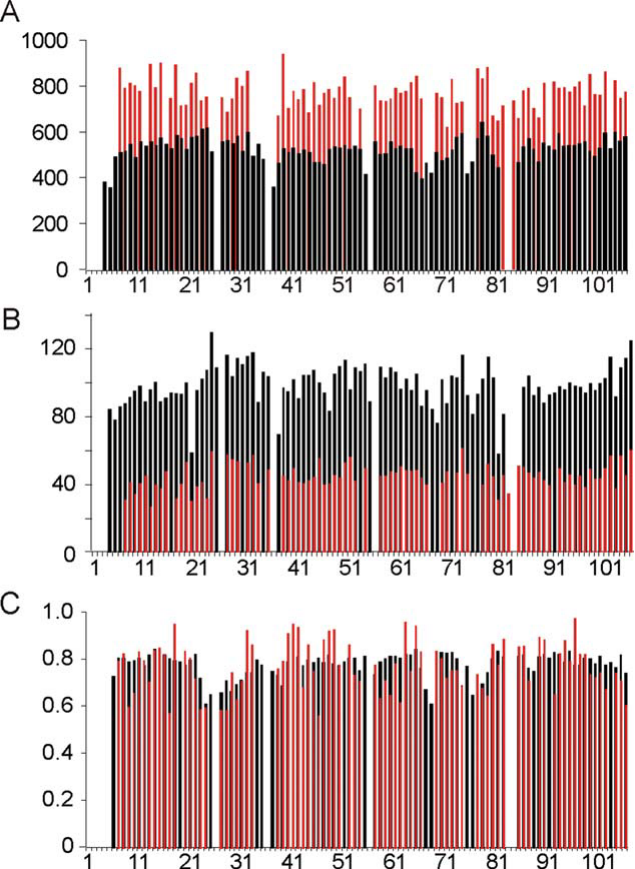
NMR relaxation parameters of Cyay in 25 m*M* Tris-HCl buffer at pH 8.3 (black bars) and in HEW (red bars) as measured at 600 MHz and 25°C. **A**: *T*_1_ values, **B**: *T*_2_ values, and **C**: NOE values.

Overall, the longitudinal (*T*_1_) and transverse (*T*_2_) relaxation rates, and the NOE values are rather uniform along the protein sequence, in agreement with what is expected for a compactly folded globular protein (Fig. 3). However, there is a clear lengthening of *T*_1_ and a shortening of *T*_2_ *in ovo* (red bars) as compared with the data in buffer, whereas no substantial difference is observed in the NOE values. The average value of *T*_1_ goes from 602 ms in buffer to 775 ms in egg white, whereas the corresponding values of *T*_2_ vary from 121 to 45 ms, respectively.

The overall rotational correlation time (τ_c_) of CyaY at room temperature in HEW, as estimated from *T*_1_/*T*_2_ ratios, is 13.1 ns. For comparison, the value in diluted buffer calculated with the same approach is 6.4 ns. Although the latter value is in excellent agreement with what is expected for a monomeric globular domain of equivalent size,^22^ the value in HEW is appreciably longer. Consistent values were independently confirmed also by a recently devised, more rapid method for the direct determination of the ^15^N *T*_1_/*T*_2_ ratios (Kelly and Frenkiel, in preparation). This method estimates the correlation time from a series of 1D profiles, in a manner conceptually similar to the TRACT method,^23^ making the estimation of correlation times faster. Also according to this method, the rotational correlation time of CyaY doubles, going from ∼6 ns in the dilute buffer solution to ∼12 ns in the crowded environment of HEW.

The rate of rotational relaxation is commonly explained in terms of solvent viscosity. The dependence of the correlation time (τ_c_) from viscosity (η) is given by the well-known Debye–Stokes–Einstein equation.^24^ According to the proportionality between τ_c_ and η, we could expect an increase of τ_c_ up to four times in going from a dilute buffer solution (with a viscosity close to 1 cp) to the thin portion of HEW, whose viscosity has been estimated as 4 cp.^25^ The doubling of τ_c_ observed experimentally, albeit smaller than the maximum possible increase, is consistent with the influence of viscosity as the main effect.

### Protein stability in a crowded environment

**Figure 4:**
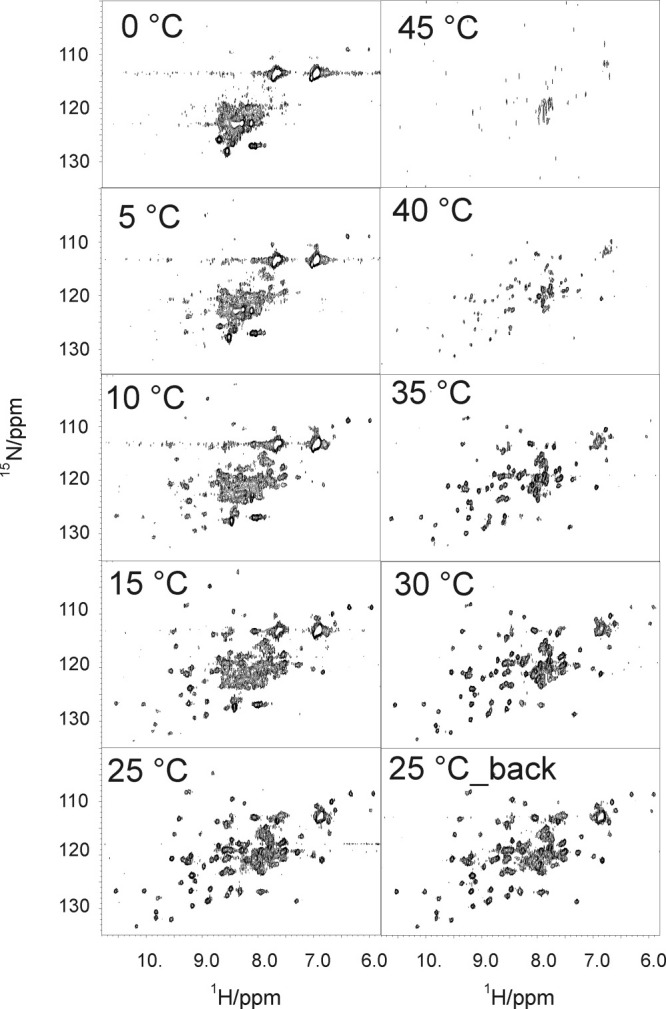
^15^N HSQC spectra of Yfh1 in HEW in a temperature range covering both low- and high-unfolding transitions.

To check the effect of HEW on protein stability, we used the yeast ortholog of CyaY, Yfh1. One of the peculiarities of this protein is that it undergoes cold denaturation at temperatures above the freezing temperature of water and heat denaturation well below the coagulation temperature of HEW.^13^,^14^,^21^ This gave us the unique opportunity to check the thermal stability of a protein in HEW over the entire range of temperature comprising heat and cold denaturation. Both HSQC spectra (Fig. 4) and relaxation behavior of this protein in HEW (Fig. S1 of Supporting Information) are comparable to those found for its bacterial ortholog.

We ran spectra of Yfh1 in the temperature range 0°C to 45°C and observed both cold and heat denaturation (Fig. 4). Although the spectral quality prevents an accurate quantitative assessment of the thermodynamic parameters of the transition, it is very interesting to observe that the temperature dependence in HEW parallels that in dilute conditions. Altogether, it is fair to say that the thermal stability of Yfh1 in HEW is not higher than thatmeasured in dilute buffer, with a low temperature transition between 5 and 10°C and a high temperature transition temperature between 30 and 35°C. As for CyaY, the spectrum of the folded species at 25°C in HEW has broadened peaks with respect to that in dilute buffer solution. It does otherwise correspond well to the one at room temperature in Hepes previously reported by us.^14^ The same can be said for the cold denatured species: the spectrum in HEW at 0°C (upper left panel in Fig. 4) is very similar to that in Hepes.^14^ On the contrary, the spectra monitoring heat denaturation in HEW look intrinsically different with respect to those in Hepes. The spectrum at 45°C in HEW, (upper right panel of Fig. 4), shows only very few barely visible peaks, whereas the published spectrum at the same temperature, in buffer, is nearly indistinguishable from that of the cold denatured species.^14^ At first sight, such a behavior seems to speak of an irreversible precipitation of the unfolded species at high temperature. However, as shown in the right bottom panel of Figure 4 (spectrum labeled 25°C_back), the sample cooled down from 45 to 25°C has a spectrum identical to the starting one, a clear indication that also the high temperature unfolding in HEW, is a completely reversible process.

The disappearance of all cross peaks parallels the unfolding of Yfh1 as the temperature is raised from room temperature to 45°C. All cross peaks reappear as the protein folds back to its native state. Two possible explanations come to mind: an enhanced exchange rate due to the higher pH in HEW or a weak interaction of the unfolded species with HEW proteins, enhanced by high temperature. We checked the behavior of Yfh1 at a pH higher than that used in the published study^14^ (pH 7.0 in 20 m*M* HEPES). It is possible to see (in Fig. S2 of Supporting Information, showing representative spectra in the range 5 to 35°C) that even at pH 8.5 (in 20 m*M* TAPS) the spectrum of the unfolded species does not disappear at high temperature. Accordingly, we favor the hypothesis based on weak interactions with HEW proteins. We hypothesize that the binding of the high-temperature unfolded species to larger HEW proteins leads to broadening of its resonances so large as to make them unobservable. These interactions, possibly due to hydrophobic forces, weaken as the temperature is decreased (and the protein concomitantly refolds). The absence of a similar behavior at low temperature is consistent with the strong hydration of the cold denatured species, we have recently described.^26^ It is fair to hypothesize that hydration of amide groups and possibly also of side chains, as implied in the standard model of cold denaturation,^27^ can effectively shield the unfolded species from weak interactions with the proteins of HEW.

## DISCUSSION

The importance of studying proteins in their cellular environment has become increasingly compelling over the last few years. It would be obviously ideal to study each protein in its own natural medium, but this choice is often precluded in structural studies by practical limitations. Most studies have, thus, tried to mimic natural crowded environments using single, purified macromolecules, not only proteins of several different molecular weights but also polysaccharides, like ficoll, or synthetic polymers, like polyethyleneglycol.^7^

In this study, we have looked for a simple natural medium, endowed of the complexity of crowded cells, yet freely available, and possibly inexpensive. The albumen of avian eggs, notably HEW, does possess all these features. The only potential drawback of HEW as a general medium to study proteins might be a time-dependent pH drift, which, however, occurs almost exclusively between the time of egg deposition and the preparation of HEW, whereas the pH is fairly stable during the time required for NMR measurements.

By studying the structural, dynamical, and stability properties of model proteins, we have demonstrated that HEW does not interfere with the structural integrity of representative natural globular proteins, such as members of the frataxin family: the folds of both tested proteins remain practically invariant. Notably, the only visible effects on the NMR spectrum of CyaY come from the presence in HEW of cations that have weak interactions with this protein, consistently with the variability of natural environments as opposed to solutions of artificial crowders. We have also found that HEW does not affect significantly protein stability but has a large influence on the overall protein tumbling time.

At first sight, the negligible effect of crowding in HEW on protein stability may seem surprising, particularly if one recalls the moderate stabilization obtained for proteins embedded in polyacrylamide gels^28^ or, even more, the spectacular increase in unfolding temperatures of proteins trapped in silica matrix.^29^ However, both represent applications of confinement rather than crowding: the former limits the volume accessible to a molecule in a static way, whereas the latter reduces the accessible volume in a dynamic way. The main difference between confinement in silica gels is that the size of the protein-occupied pores in silica is dictated by the protein itself.^30^ As a consequence, the matrix impedes almost completely the rotational freedom of the protein^31^ with dramatic effects on protein stability. Although early studies on crowding foresaw sizeable effects on folding, the influence of crowding on protein stability has now been demonstrated to be small. The main reason for predicting large effects relied on modeling the folded form of a protein as a unique globular sphere, whereas the excluded volumes of unfolded proteins were represented as beads in a pearl necklace.^32^ This model predicted that crowding should greatly favor the folded state over the unfolded one. Minton^33^ challenged this vision and some of its consequences, by proposing that the volume excluded by the denatured state can rather be likened to the convex envelope of a Brownian walk. As a consequence, the volumes of crowding molecules excluded by folded and unfolded species are comparable. Experimental studies on macromolecular crowding have confirmed a modest effect on protein stability,^34^ thus, supporting earlier theoretical predictions.^33^,^35^ On the contrary, both simplified models^33^,^35^ and experimental findings^28^,^29^,^36^,^37^ have shown that confinement stabilizes proteins and may accelerate their folding significantly.

The influences of different crowding agents on rotational diffusion^10^ and on the effects of volume exclusion^7^ of a small protein have been recently investigated. These authors suggested that the difference between the behavior of synthetic polymers and protein crowders could be attributed to the presence of weak interactions between the crowding proteins and the small probe protein. These authors observed that using large proteins as crowding agents (instead of synthetic polymers) can lead to the disappearance of the NMR HSQC spectra of folded proteins, as a result of weak interactions with the crowding agents. Our observation of the disappearance of the spectrum of the unfolded species after the high-temperature transition is in line with the results of Wang *et al*.^10^ Interestingly, the main component of HEW, that is, ovalbumin, is also one of the crowder proteins used by Wang *et al*. in their study. However, we have not observed any dramatic effect of HEW on the HSQC spectra of folded CyaY or Yfh1.

Taken together, our results strongly support the use of HEW as an excellent medium with which to study the effect of crowding without interference of confinement effects. HEW is also a unique medium to exploit by NMR, because labeling, a procedure routinely applied instructural NMR studies of proteins, effectively lets us to study desired probe proteins in a sea of other molecules which, being unlabeled, are “transparent” to the observer. It is possible to foresee that HEW will be very valuable in studying aggregation phenomena because they should be greatly enhanced under crowding conditions. These studies are currently in progress in our laboratory.
