# Supplementary material for: A natural and readily available crowding agent: NMR studies of proteins in hen egg white
Source: Proteins. 2010 Dec 13;79(5):1408–15. doi: 10.1002/prot.22967 (PMC3110865; doi:10.1002/prot.22967)
Supplement: Supplementary file 1 [file prot0079-1408-SD1.pdf]

Supplementary Online Material

A natural and easily available crowding agent: NMR studies of proteins in hen egg white

Gabriel Martorell[a], Miquel Adrover[b,c], Geoff Kelly[b], Piero Andrea Temussi[b,d], Annalisa Pastore[b]*

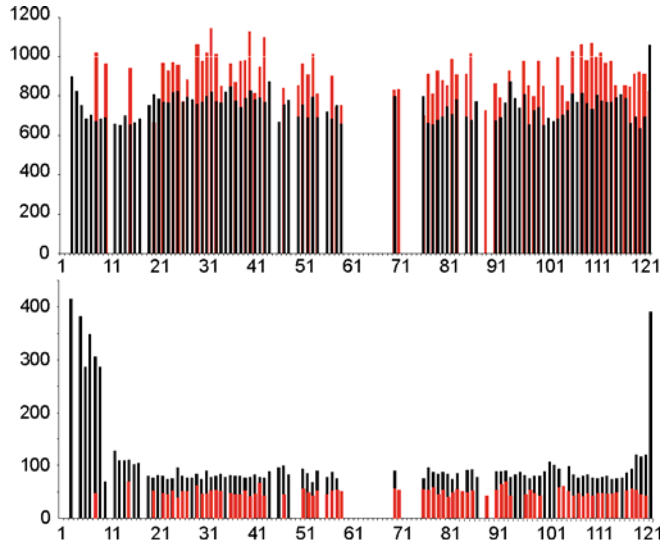

Figure S1. NMR relaxation parameters of Yfh1 in 25 mM Tris-HCl buffer at pH 8.3 (black bars) and in HEW (red bars) as measured at 600 MHz and 25°C. A) T1 values B) T2.

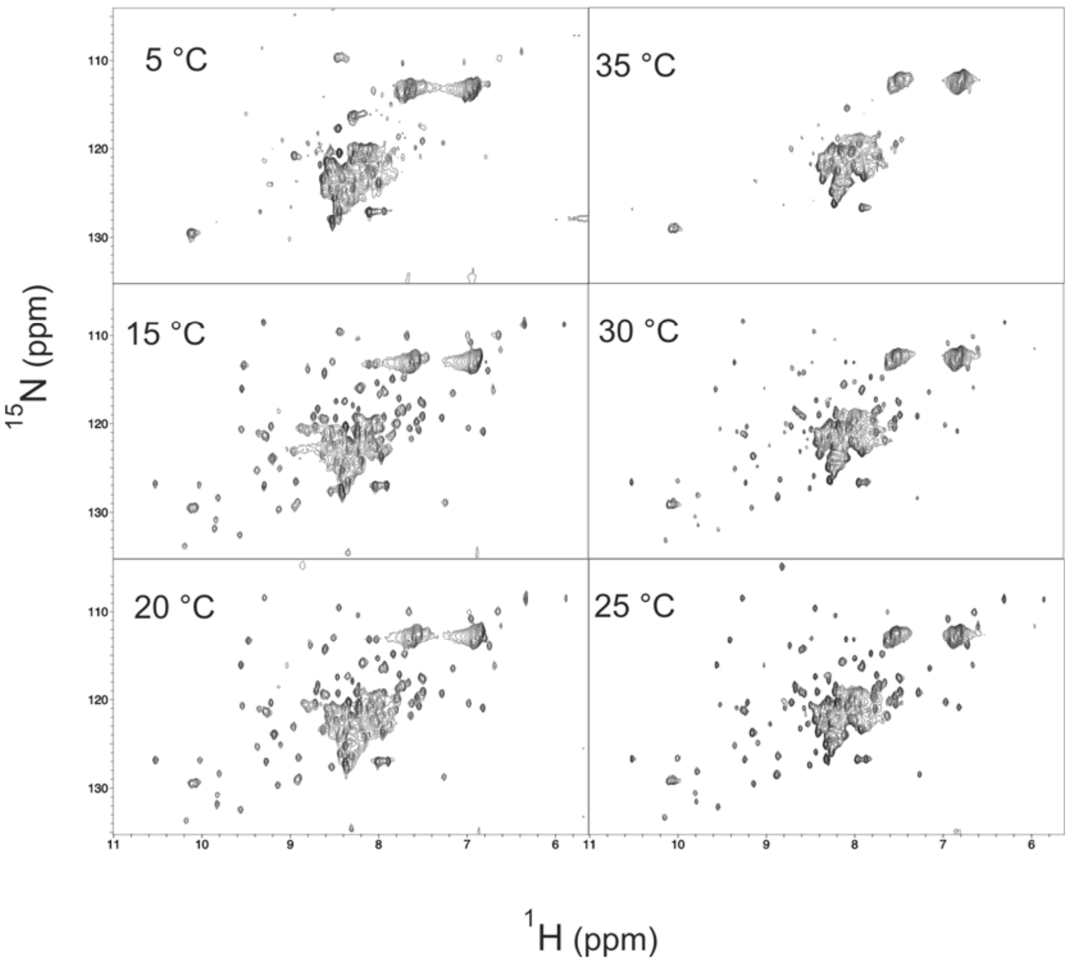

Figure S2. Partial temperature dependence of Yfh1 at basic pH. ^{15}N HSQC spectra of Yfh1 at pH 8.5 (in 20 mM TAPS) in a temperature range covering both low and high unfolding transitions. The high temperature transition occurs at lower temperature than at pH 7, but the spectrum of the unfolded species does not disappear.
